# Effectiveness of exercise interventions on urinary incontinence and pelvic organ prolapse in pregnant and postpartum women: umbrella review and clinical guideline development

**DOI:** 10.1097/XEB.0000000000000391

**Published:** 2023-10-18

**Authors:** Iina Ryhtä, Anna Axelin, Heidi Parisod, Arja Holopainen, Lotta Hamari

**Affiliations:** 1 Department of Nursing Science, Faculty of Medicine, University of Turku, Turku, Finland; 2 Department of Obstetrics and Gynaecology, Turku University Hospital, Turku, Finland; 3 Nursing Research Foundation, Helsinki, Finland; 4 The Finnish Centre for Evidence-Based Health Care: JBI Centre of Excellence, Helsinki, Finland; 5 WHO Collaborating Centre for Nursing, Helsinki, Finland

**Keywords:** exercise, maternal health, pelvic floor, pelvic organ prolapse, postpartum, pregnancy, urinary incontinence

## Abstract

**Introduction and aims:**

Dysfunction related to pelvic floor muscles (PFM) is common among pregnant and postpartum women and can cause symptoms such as urinary incontinence or pelvic organ prolapse (POP). As part of developing a nationwide clinical practice guideline for nursing in Finland, the aim of this umbrella review is to summarize the existing evidence about the effectiveness of exercise interventions on urinary incontinence and POP in pregnant and postpartum women. To promote knowledge translation, recommendations for health care professionals are presented.

**Methods:**

We conducted an umbrella review to summarize the existing evidence. The JBI methodology for umbrella reviews was used to guide the review. The level of evidence was evaluated using the Grading of Recommendations, Assessment, Development and Evaluation (GRADE) approach, and recommendations based on the evidence were formulated by a clinical guideline working group.

**Results:**

Altogether, 9 systematic reviews, reporting findings from 89 original studies, were included. The methodological quality of the reviews was evaluated using JBI's checklist. The highest level of evidence was found for preventing the symptoms of postpartum urinary incontinence through exercise and pelvic floor muscle training (PFMT) during pregnancy. Moderate-level evidence showed that exercise and PFMT are likely to reduce the symptoms and severity of urinary incontinence, but the level of evidence was low on PFMT reducing the symptoms of POP.

**Conclusion:**

We recommend encouraging and guiding pregnant and postpartum women to exercise and train PFM. We also recommend identifying pregnant and postpartum women with symptoms of PFM dysfunction and directing them to a physiotherapist or other health care professional specializing in pelvic floor function.

**What is known about the topic?**

- Dysfunction related to pelvic floor muscles is common among pregnant and postpartum women and can cause symptoms such as urinary incontinence and pelvic organ prolapse, which can severely decrease the well-being of women.
- The practices for guiding pregnant and postpartum women to physical activity and exercise vary in Finland, and national clinical guidelines on this topic are lacking.
- Recommendations are also lacking for health care professionals on when to refer pregnant and postpartum women to more individualized pelvic floor muscle assessment and guidance.

**What does this article add?**

- This article bridges evidence synthesis and evidence transfer through an umbrella review. It provides recommendations for health care professionals on guiding pregnant and postpartum women.
- Based on the synthesized evidence, women who already have pelvic floor dysfunction during pregnancy or postpartum should be guided to a physical therapist or other health care professional who specializes in the pelvic floor.
- This article provides a possibility for international audiences to understand and replicate the process by which Finnish national evidence-based clinical guidelines for nursing are developed.

## Introduction

Pregnancy and childbirth change the bodies of women, and some of the most notable anatomical changes occur in the pelvic area. The progressive increase in volume of the uterus causes a major overload in perineal structures, commonly resulting in muscle trauma, nerve injuries, and connective tissue damage.^1,^^2^ Pelvic floor muscles (PFM) support the bladder, vagina, rectum, and lower back.^3^ Reduced strength and functionality of PFM can cause dysfunction of the PFM, resulting in different common and bothersome symptoms^2,^^4^ such as urinary incontinence^5^ or pelvic organ prolapse (POP).^6^

Urinary incontinence refers to the unintentional passing of urine,^7^ with prevalence varying from 30% to 70%.^1,^^5,^^8,^^9^ Typically, urinary incontinence increases during pregnancy and gradually decreases during the first postpartum year, with prevalence varying from 30% to 58%.^9,^^10^ POP refers to the bulging of the uterus, bladder, and/or bowel as a result of impairment of the supporting tissues surrounding the vagina.^11^ The prevalence of POP in women varies from 3% to 50%,^12,^^13^ and it is known that pregnancy and childbirth are risk factors for developing POP.^14^ The symptoms of POP can severely decrease the quality of life of women,^15^ and health care professionals working with pregnant and postpartum women need evidence-based knowledge to guide and support women to prevent and care for the symptoms of urinary incontinence and POP.

It is common that the amount of exercise decreases as the pregnancy progresses and only a few pregnant women move as much as recommended.^16,^^17^ Physical factors related to pregnancy, such as pain, swelling, or nausea may be the causes of reduced exercise, as well as concerns about pregnancy complications. Moreover, lack of motivation, knowledge, and guidance may be the causes of reduced exercise.^18^ It is also common that after birth, the amount of exercise may not reach the desired level or return to pre-pregnancy levels.^19^

In Finland, all pregnant women are entitled to free maternity and child health services during pregnancy and after birth. During pregnancy, there are usually 11–15 visits, mainly not only with public health nurses but also with doctors. The purpose of the visits is to follow up on the growth and development of the fetus and the health of the woman during pregnancy and after birth.^20^ Public health nurses should provide guidance for pregnant and postpartum women, for example, regarding exercise.

This review was conducted as part of the development of the Finnish national clinical practice guidelines, “Pregnant and postpartum women in health care: justification and guidance for physical activity and exercise,” coordinated by the Nursing Research Foundation (NRF).^21^ The guidelines coordinated and published by the NRF are evidence-based recommendations mainly for nursing professionals. The process of developing guidelines includes 11 phases. Development of the guidelines starts with receiving and accepting the suggested topic and preparing the work plan (phases 1–3). The next phases are systematic literature search; methodological quality assessment of the included studies; rating the level of evidence based on the Grading of Recommendations, Assessment, Development and Evaluation (GRADE) approach; formulating the recommendations; and external expert review (phases 4–8). The last phases are publication and dissemination of the guidelines; implementation; and updating the guidelines (phases 9–11).^22^

**Figure 1:**
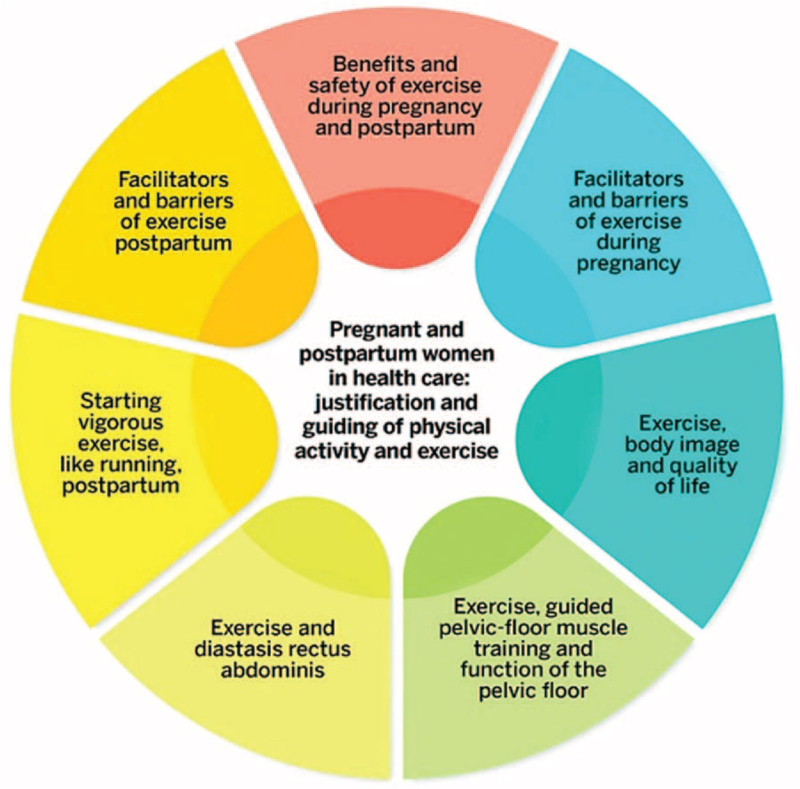
Nursing Research Foundation guideline, “Pregnant and postpartum women in health care: justification and guidance for physical activity and exercise”.

The guideline “Pregnant and postpartum women in health care: justification and guidance for physical activity and exercise” includes seven sections. This umbrella review forms the basis of one section of the guideline: “Exercise, guided pelvic floor muscle training and function of the pelvic floor” (see Figure 1).

The aim of this umbrella review was to summarize the existing evidence about the effectiveness of exercise interventions on urinary incontinence and POP in pregnant and postpartum women. The aim of the guidelines is to unify the exercise guidance given to pregnant and postpartum women in maternity and child health services in Finland, and to reduce any unwarranted variations in guidance.

## Review questions

(1) What is the effectiveness of exercise interventions on urinary incontinence in pregnant and postpartum women?
(2) What is the effectiveness of exercise interventions on POP in pregnant and postpartum women?

## Methods

We conducted an umbrella review to form an overall understanding on the topic of interest and to identify areas for future research.^23^ We followed the JBI Manual for Evidence Synthesis^23^ and the Preferred Reporting Items for Systematic Reviews and Meta-Analyses (PRISMA) guidelines.^24^ The protocol produced *a priori* was registered in PROSPERO (CRD42020191591). After rating the level of evidence, recommendations were formulated by a clinical guideline working group based on the guideline handbook of the NRF.^22^

### Inclusion criteria

Reviews were included if they met the following criteria: (i) types of study: systematic review and/or meta-analysis of effectiveness; (ii) population: pregnant and/or postpartum women up to 24 months from birth. According to Romano *et al.*,^25^ the postpartum period lasts until 6 months after birth, but in this review, the time frame was set at 24 months so that the long-term effectiveness of the interventions could be identified; (iii) interventions: any type of physical activity (e.g. exercise, physiotherapy) or guidance to address the symptoms of UI and/or POP, delivered individually, in a group, face-to-face, or online; (iv) comparison: usual care, no exercise, or being on a waiting list; (v) outcomes: symptoms of urinary incontinence and/or POP; and (vi) language: English or Finnish, as there was no funding for translations.

### Search strategy and data sources

Searches were conducted in MEDLINE (PubMed), CINAHL (EBSCOhost), PsycINFO (EBSCOhost), Web of Science, Medic, Cochrane Library, ERIC (EBSCOhost), Embase, and Academic Search Premier (EBSCOhost) on 3 January 2022. A search update was conducted on 11 January 2023. The search strategy followed the PICO model.^26^ Detailed search strategies are available in Appendix I, http://links.lww.com/IJEBH/A138. The search was conducted with the assistance of an information specialist.

### Screening and selection

**Figure 2:**
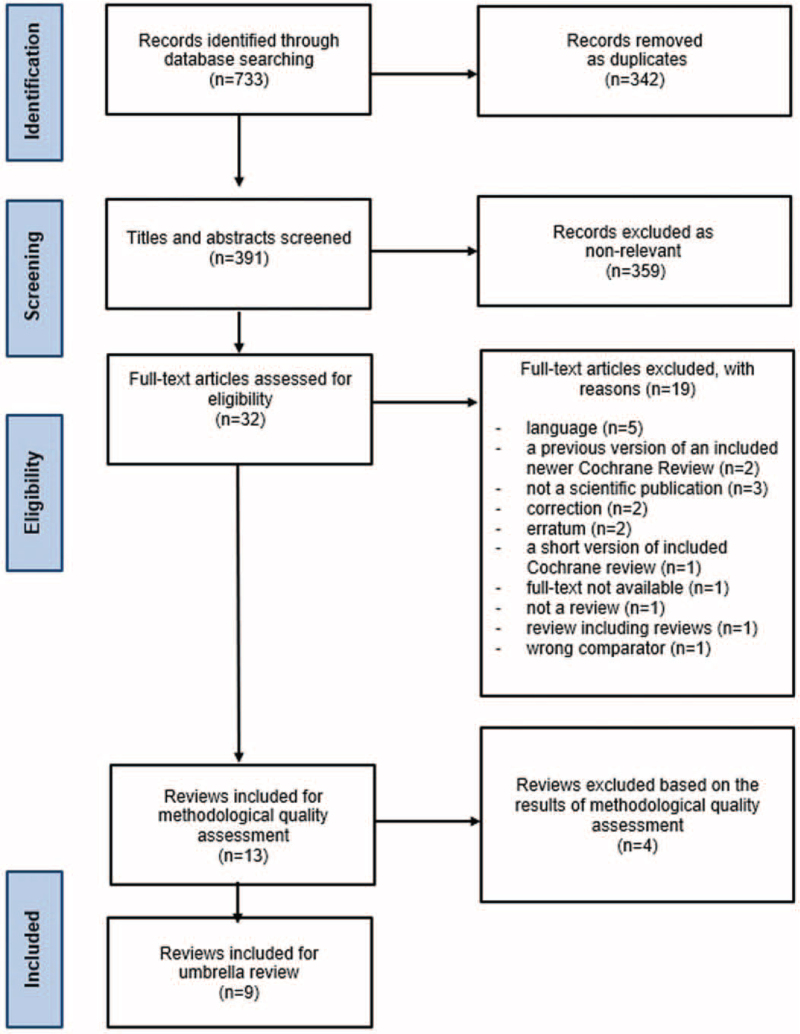
PRISMA flow chart of screening process.

First, two authors (IR, LH) independently screened the titles and abstracts by applying the established eligibility criteria, and after that, any conflicts were discussed to reach consensus. The full texts were screened similarly. The decisions in all phases were recorded using Covidence software (Veritas Health Innovation, Melbourne, Australia). The study selection process is described in a PRISMA flowchart (see Figure 2).

### Critical appraisal

Two authors (IR, LH) independently performed the methodological quality assessment of the reviews using the JBI Critical Appraisal Checklist for Systematic Reviews and Research Syntheses.^27^ If the information was not available or was unclearly stated, authors contacted the corresponding authors of these articles. Disagreements over assessments were resolved through discussion. The checklist consists of 11 items (see Table 1) with 4 possible responses: *Yes* (the criterion is clearly identifiable and assigned 1 point), *No* (the criterion failed to be applied appropriately), *Unclear* (the criterion is not clearly identified in the report), and *Not applicable.* It was determined *a priori* that the overall scores of a review needed to be at least 6/11 to be included in this umbrella review. Moreover, Item 5: *Critical appraisal of studies*, needed to have a *Yes* answer. This was done to ensure the quality of the included reviews.^22^

### Data extraction and analysis

Two authors (IR, LH) independently carried out the data extraction according to JBI's instructions,^23^ using Covidence. The data extraction table was tested before its actual use. The analysis of the included reviews was done narratively. The summaries of findings from the included reviews were presented in a table.

### Grading the level of evidence and forming the recommendations

If the GRADE assessments were already done by the authors of the included reviews, the same gradings were used. If the GRADE ratings varied for different reviews, the methodological quality ratings of the reviews, publication year, and clinical relevance of the results were considered (emphasis was placed on the most recent, highest quality systematic reviews and clinical relevance). Regarding the level of evidence and the wording of the recommendations, the NRF guideline handbook was followed. The level of evidence is interpreted as follows: A = high, B = moderate, C = low, and D = very low.^22^

## Results

### Study inclusion

After duplicates (*n* = 342) were removed, 391 titles and abstracts were screened. After excluding irrelevant records (*n* = 359), 32 full-text articles were assessed for eligibility, following which a further 19 articles were excluded. Consequently, 13 articles were included for the methodological quality assessment; however, 4 were excluded because of poor quality. As a result, 9 articles were included in the umbrella review, forming the basis of the recommendations (see Figure 2). The list of excluded studies is available from the authors upon request.

### Methodological quality

The methodological quality of the reviews was assessed using JBI's Critical Appraisal Checklist for Systematic Reviews and Research Syntheses.^27^ Four studies scored the maximum 11/11 points^7,^^28–^^30^ (see Table 1).

**Table 1:** Methodological quality of included reviews

	Items^a^											
Review (*n* = 9)	1	2	3	4	5	6	7	8	9	10	11	Points
von Aarburg *et al.*, 2021^31^	Yes	No	Yes	Yes	Yes	Yes	Yes	Yes	No	Yes	Yes	9/11
Davenport *et al.*, 2018^28^	Yes	Yes	Yes	Yes	Yes	Yes	Yes	Yes	Yes	Yes	Yes	11/11
Harvey, 2003^32^	No	No	No	Yes	Yes	No	Yes	Yes	No	Yes	Yes	6/11
Lemos *et al.*, 2008^33^	Yes	Yes	Yes	Yes	Yes	Yes	No	Yes	No	Yes	Yes	9/11
Mørkved and Bø, 2014^34^	Yes	Yes	Yes	Yes	Yes	Yes	No	Yes	No	Yes	Yes	9/11
Wagg and Bunn, 2007^35^	No	No	No	Yes	Yes	Yes	Yes	Yes	No	Yes	Yes	7/11
Woodley *et al.*, 2020^7^	Yes	Yes	Yes	Yes	Yes	Yes	Yes	Yes	Yes	Yes	Yes	11/11
Wu *et al.*, 2018^29^	Yes	Yes	Yes	Yes	Yes	Yes	Yes	Yes	Yes	Yes	Yes	11/11
Yang *et al.*, 2022^30^	Yes	Yes	Yes	Yes	Yes	Yes	Yes	Yes	Yes	Yes	Yes	11/11

a Items from the JBI Critical Appraisal Checklist for Systematic Reviews and Research Syntheses^27^: 1. Is the review question clearly and explicitly stated? 2. Were the inclusion criteria appropriate for the review question? 3. Was the search strategy appropriate? 4. Were the sources and resources used to search for studies adequate? 5. Were the criteria for appraising studies appropriate? 6. Was critical appraisal conducted by 2 or more reviewers independently? 7. Were there methods to minimize errors in data extraction? 8. Were the methods used to combine studies appropriate? 9. Was the likelihood of publication bias assessed? 10. Were recommendations for policy and/or practice supported by the reported data? 11. Were the specific directives for new research appropriate?

### Characteristics of included studies

The 9 included reviews, consisting of 89 original studies, were published between 2003 and 2022 (see Appendix II, http://links.lww.com/IJEBH/A139 for a list of original studies). All were systematic reviews, of which 4 were meta-analyses, 2 were narrative analyses, and 3 were both meta-analyses and narrative analyses. The main purpose of the reviews was to assess the effectiveness of exercise interventions in pregnant (*n* = 2), postpartum (*n* = 2), or both pregnant and postpartum women (*n* = 5). Primary outcomes assessed were urinary incontinence (*n* = 8) and POP (*n* = 1) (see Table 2).

**Table 2:** Characteristics of included reviews (*n* = 9) and interventions

Primary outcome UI (*n* = 8)						
Review, year (country)	Primary outcome	Interventions used	Total number/sample size, participants	Number of original studies in review	Methodological quality, appraisal tool	Method of analysis
von Aarburg *et al.*, 2021^31^ (Switzerland)	Incidence of UI	F: not reported I: not reported T: not reported Ty: walking, brisk walking, running, bicycling/spinning, strength training, weight training, gymnastics, jumping, swimming, aerobics (low and high impact), aerobic dance/step, prenatal aerobic classes, dancing, Pilates, cross-country skiing/roller skiing, ball games, horseback riding, skating/rollerblades S: pregnancy follow-ups or postpartum consultations in hospitals or clinics, sports organizations	12 479/40–10 418, pregnant and postpartum women	*n* = 7 (*n* = 6 observational studies, *n* = 1 experimental study)	Low quality, Cochrane Risk of Bias tool	Meta-analysis and narrative synthesis
Davenport *et al.*, 2018^28^ (Canada)	Prevalence and severity of UI	F: ranged from 1 to 7 times per week I: ranged from light to moderate T: duration of interventions was not reported. Interventions were initiated between 9 and 30 GWs. Ty: aerobic exercise, PFMT S: at clinics and home, both independently and/or supervised	15 982/64–10098, pregnant women	*n* = 24 (*n* = 18 RCTs, *n* = 2 quasi-experimental studies, *n* = 4 cohort studies)	Not reported, Cochrane Risk of Bias tool	Meta-analysis and narrative synthesis
Harvey, 2003^32^ (Canada)	Incontinence, prolapse, pelvic floor strength	F: not reported in all studies, ranged from 3 to 7 times per week I: not reported in all studies, one study reported training intensity being 60–70% maximal heart rate T: one study reported duration of intervention being 12 weeks Ty: pelvic floor training, Kegel contractions, aerobic exercise S: at home and in clinics, direct teaching and supervision of the technique by experienced physiotherapists, both individually and in groups	3573/45–1169, pregnant and postpartum women	*n* = 9 RCTs	Between poor and good quality (*n* = 7) good quality (*n* = 2), Jadad scale	Meta-analysis and narrative synthesis
Lemos *et al.*, 2008^33^ (Brazil)	Prevalence of UI	F: ranged from 1 to 10 times a day I: varied T: duration of interventions varied from 16 to 20 weeks. Interventions were initiated in the 20 GW. Ty: PFMT S: at home	675/72–301, pregnant women	*n* = 4 RCTs	High quality (*n* = 4), Jadad scale	Meta-analysis
Mørkved and Bø, 2014^34^ (Norway)	UI	F: varied I: varied; strong (near maximal) contractions T: duration of interventions varied from 12 to 20 weeks. Interventions were initiated between 11 and 24 GWs. Ty: PFMT S: both regular home training and follow-up (monthly and weekly) by a physical therapist	3731/not reported, pregnant and postpartum women	*n* = 22 (*n* = 10 RCTs, *n* = 2 quasi-experimental studies)	Scores between 3 and 8/10, mainly good quality, PEDro scale	Narrative synthesis
Wagg and Bunn, 2007^35^ (UK)	UI	F: not reported I: not reported T: not reported Ty: PFMT S: interventions were led by nurses, midwives, or physiotherapists and conducted individually or in groups	4380/72–1800, postpartum women	*n* = 6 RCTs	Quality varied	Narrative synthesis
Woodley *et al.*, 2020^7^ (New Zealand)	UI, FI, incontinence-specific QoL	F: ranged from 1 to 3 times per week to >2 times per day I: mainly not reported, one study reported progressive PFMT with increased intensity every week T: duration of interventions ranged from 8 weeks to 17 months Ty: strength training, physical conditioning program, aerobic fitness, stretching, stabilization exercises, Kegel exercises, PFMT S: at home and/or in exercise class, independently and/or under supervision by a physiotherapist, midwife, nurse, or other qualified person in hospitals	10 832/20–1800, pregnant and postpartum women	*n* = 46 (RCTs and quasi-experimental studies)	Low to moderate quality, Cochrane Risk of Bias tool	Meta-analysis
Yang *et al.*, 2022^30^ (UK)	UI	F: home exercises twice a day, in one study not reported, group sessions from once a week to once a month I: not reported T: duration of interventions ranged from 6 weeks to 4 months. Interventions were initiated between 16 GWs to 6 weeks after birth. Ty: PFMT S: training was instructed by physiotherapists or midwives in groups	1132/70–301, pregnant and/or postpartum women	*n* = 5 RCTs	Low quality, Cochrane Risk of Bias tool	Meta-analysis

Primary outcome POP (*n* = 1)						
Review, year (country)	Primary outcome	Interventions used	Total number/sample size, participants	Number of original studies in review	Methodological quality, appraisal tool	Method of analysis
Wu *et al.*, 2018^29^ (Canada)	POP symptoms	F: not reported I: not reported T: duration of interventions varied from 4 weeks to 9 months. Interventions were initiated between 1 week postpartum to 12 months after birth, but most interventions were initiated at 6–8 weeks after birth. Ty: structured pelvic floor muscle exercises S: taught and supervised by trained personnel	3845/not reported, postpartum women	*n* = 15 RCTs	Low to moderate quality, Cochrane Risk of Bias tool	Meta-analysis

F, frequency; FI, fecal incontinence; GW, gestational week; I, intensity; PFMT, pelvic floor muscle training; POP, pelvic organ prolapse; QoL, quality of life; RCT, randomized controlled trial; S, setting; T, time; Ty, type; UI, urinary incontinence.

The interventions and the degree of description of the interventions in the reviews were heterogeneous. The exercise protocols varied from solely pelvic floor muscle training (PFMT)^29,^^30,^^33–^^35^ to combinations of aerobic exercise and PFMT.^28,^^32^ In 2 reviews, the exercise interventions included various sports, for example, strength training, stretching, or aerobic exercise.^7,^^31^ The exercise interventions were initiated independently and/or in groups; and/or under supervision of a physiotherapist, midwife, nurse, or other qualified person; in hospitals, exercise classes, or at home. The frequency of exercise varied from several times a day to once a week and the duration of the intervention was from 4 weeks to 17 months. The interventions began between gestational week 9 and 12 months postpartum. The interventions were described according to FITT principles (frequency, intensity, time, type).^36^ In addition, the setting of the intervention was added (see Table 2).

### Summary of findings

#### Exercise and pelvic floor muscle training during pregnancy and urinary incontinence risk and symptoms during pregnancy

A meta-analysis including 15 randomized controlled trials (RCTs) (*n* = 2764 women) found a 50% reduction in the odds of developing urinary incontinence during pregnancy between the women in the exercise intervention group and women in the control group [odds ratio (OR) 0.50, 95% confidence interval (CI) 0.37–0.68, *I*^*2*^ = 60%].^28^ Another meta-analysis of 11 RCTs (*n* = 3307 women) found that exercise and PFMT for mixed prevention and treatment of urinary incontinence during pregnancy decreases urinary incontinence risk in late pregnancy compared with women in the control group [risk ratio (RR) 0.78, 95% CI 0.64–0.94, *I*^*2*^ = 79%).^7^ In contrast, a meta-analysis of 3 observational studies (*n* = 960 women) found no statistically significant association between physical activity and urinary incontinence during pregnancy (OR 0.90, 95% CI 0.69–1.18, *p* = 0.45, *I*^*2*^ = 0%).^31^

#### Exercise during pregnancy and severity of urinary incontinence symptoms during pregnancy

A meta-analysis including 5 RCTs (*n* = 465 women) showed a moderate reduction in the severity of the symptoms of urinary incontinence during pregnancy between the exercise intervention group and control group (SMD −0.54, 95% CI −0.88 to −0.20, *I*^*2*^ = 64%).^28^

#### Exercise and pelvic floor muscle training during pregnancy and the risk and reduction of postpartum urinary incontinence symptoms

Two meta-analyses found that exercise during pregnancy decreased the risk of developing postpartum urinary incontinence (OR 0.63, 95% CI 0.51–0.79, *I*^*2*^ = 0%)^28^ (OR 0.45, 95% CI 0.31–0.66, *p* < 0,0001, *I*^*2*^ = 7%).^33^ Women performing PFMT during pregnancy for prevention (RR 0.38, 95% CI 0.17–0.83, *I*^*2*^ = 74%)^7^ and for mixed prevention and treatment (RR 0.83, 95% CI 0.71–0.99, *I*^*2*^ = 0%)^7^ of postpartum urinary incontinence were less likely to report symptoms of urinary incontinence in early postpartum (6 weeks to 3 months postpartum). In addition, PFMT during pregnancy may reduce the risk of urinary incontinence in mid-postpartum (>3 to 6 months postpartum) (RR 0.71, 95% CI 0.54–0.95, *I*^*2*^ = 0%)^7^ (RR 0.75, 95% CI 0.56–1.02, *p* = 0.028)^32^ (RR 0.73, 95% CI 0.55–0.97, *I*^*2*^ = 65%).^7^ In contrast, in late postpartum (>6 to 12 months postpartum) (RR 0.85, 95% CI 0.63–1.14, *I*^*2*^ = 0%),^7^ 12 months postpartum (RR 1.20, 95% CI 0.65–2.21),^7^ and in the long-term (>5 years) (RR 1.07, 95% CI 0.77–1.48, *I*^*2*^ = 25%)^7^, there was no evidence of a difference between women in the intervention group and the control group in terms of risk in the prevalence of urinary incontinence. A narrative analysis systematic review of 22 studies (*n* = 3731 women) found that exercise interventions during pregnancy showed a significant reduction in symptoms, episodes of urinary incontinence, or a lower percentage of women with urinary incontinence in late pregnancy or during the first 3 months after birth.^34^

#### Exercise during pregnancy and severity of postpartum urinary incontinence symptoms

A meta-analysis including 3 RCTs (*n* = 284 women) found that exercise and PFMT during pregnancy played a moderate role in reducing the severity of postpartum urinary incontinence symptoms (SMD −0.54, 95% CI −0.87 to −0.22, *I*^*2*^ = 24%).^28^

#### Pelvic floor muscle training during pregnancy and prevention of symptoms of urinary incontinence during pregnancy

Continent women performing PFMT during pregnancy had a lower risk of reporting urinary incontinence in late pregnancy (62% less, RR 0.38, 95% CI 0.20–0.72, *I*^*2*^ = 78%).^7^

#### Group pelvic floor muscle training during pregnancy and prevalence of urinary incontinence during pregnancy or postpartum

Group PFMT during pregnancy significantly reduced the prevalence of urinary incontinence during pregnancy (RR 0.67, 95% CI 0.57–0.80, *p* < 0.00001, *I*^*2*^ = 0%)^30^ and postpartum urinary incontinence (RR 0.66, 95% CI 0.52–0.84, *p* = 0.0008, *I*^*2*^ = 0%).^30^

#### Pelvic floor muscle training during pregnancy and treatment and risk of urinary incontinence during late pregnancy and postpartum

PFMT for the treatment of urinary incontinence during pregnancy was not effective and there was no evidence of any difference in the risk of urinary incontinence in late pregnancy (RR 0.70, 95% CI 0.44–1.13, *I*^*2*^ = 71%),^7^ in early postpartum (RR 0.75, 95% CI 0.37–1.53, *I*^*2*^ = 65%),^7^ in mid-postpartum (RR 0.94, 95% CI 0.70–1.24, *I*^*2*^ = 65%),^7^ or in late postpartum (RR 0.50, 95% CI 0.13–1.93, *I*^*2*^ = 94%).^7^

#### Exercise and pelvic floor muscle training postpartum and risk and symptoms of postpartum urinary incontinence

A meta-analysis including 2 observational studies (*n* = 742 women) found that there was no statistically significant association between postpartum physical activity and postpartum urinary incontinence (OR 1.31, 95% CI 0.74–2.34, *p* = 0.35, *I*^*2*^ = 42%).^31^ A meta-analysis including 2 RCTs (*n* = 321 women) found that women performing PFMT were 46% less likely to report urinary incontinence in early postpartum (RR 0.54, 95% CI 0.44–0.66, *I*^*2*^ = 0%),^7^ but no evidence of a difference in the risk of urinary incontinence in women randomized to PFMT or control group in mid-postpartum (RR 0.95, 95% CI 0.75–1.19, *I*^*2*^ = 65%),^7^ in late postpartum (RR 0.88, 95% CI 0.71–1.09, *I*^*2*^ = 50%),^7^ 6 years postpartum (RR 0.96, 95% CI 0.88–1.05),^7^ or 12 years postpartum were found (RR 1.03, 95% CI 0.94–1.12).^7^ However, 1 meta-analysis including 7 RCTs (*n* = 2692 women) found that women performing PFMT showed a significant reduction in urinary incontinence still 12 months postpartum (RR, 0.44, 95% CI 0.25–0.75, *I*^*2*^ = 91%).^29^

#### Pelvic floor muscle training postpartum and treatment of symptoms of POP postpartum

A meta-analysis including 3 RCTs (*n* = 609 women) found that structured PFMT compared with watchful waiting showed a reduction in vaginal bulging in late postpartum (RR, 0.48, 95% CI 0.30 to 0.76, *I*^*2*^ = 47%).^29^ A meta-analysis including 6 RCTs (*n* = 1275 women) showed there may be a reduction of stage II or greater POP within 12 months postpartum (RR 0.74, 95% CI 0.45 to 1.24, *I*^*2*^ = 47%).^29^

### Recommendations for clinical practice

Based on the summary of findings of this review, the recommendations were formulated by a clinical guideline working group (see Table 3). The NRF guideline handbook was followed in terms of the level of evidence and the wording of the recommendations.^22^

**Table 3:**
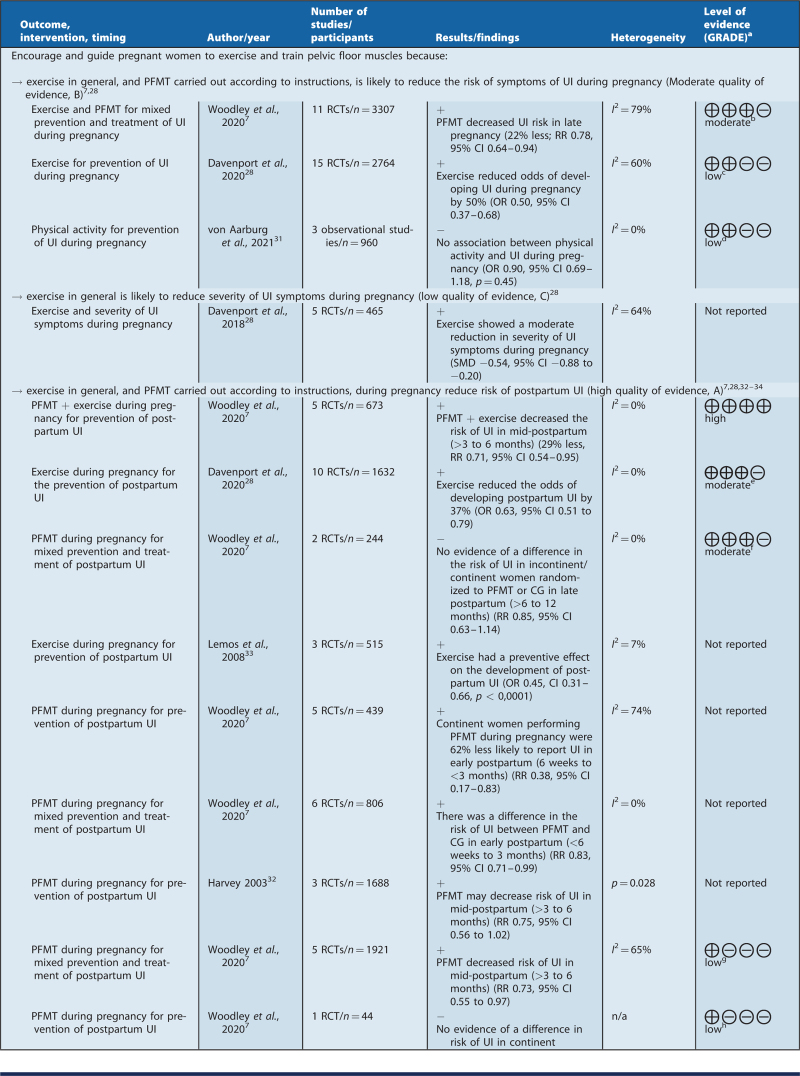
Recommendations for clinical practice and summary of findings

**Table 3(Continued):**
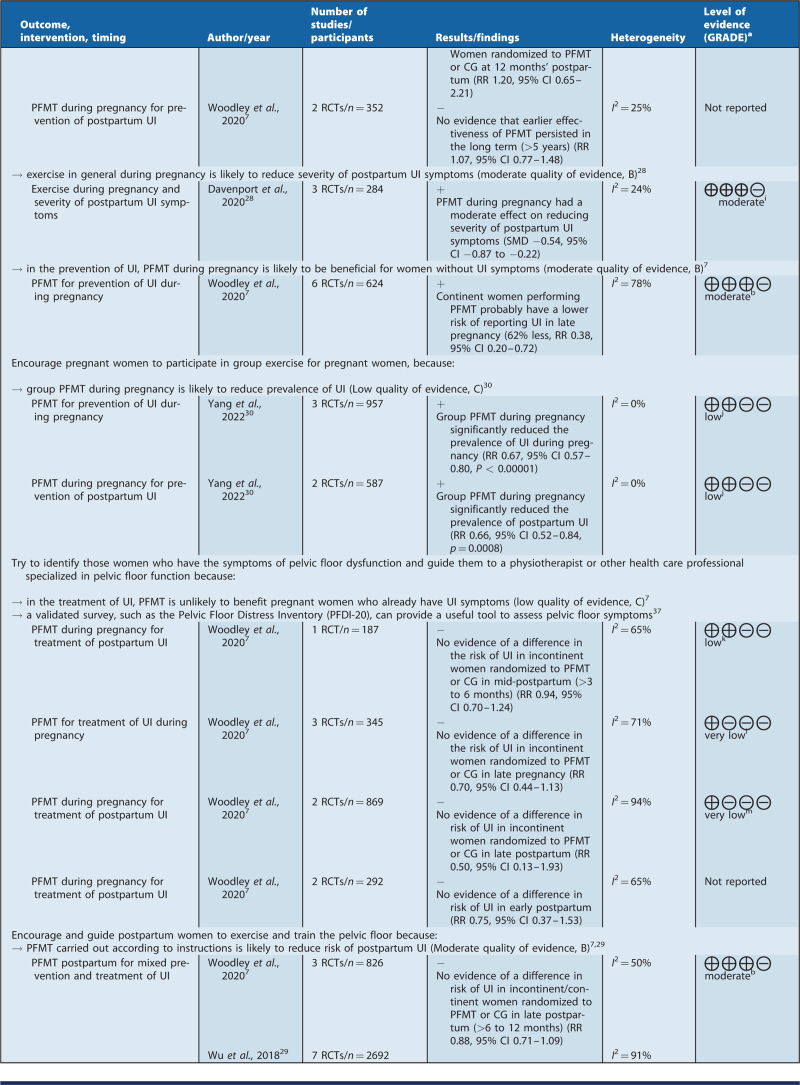
Recommendations for clinical practice and summary of findings

**Table 3(Continued):**
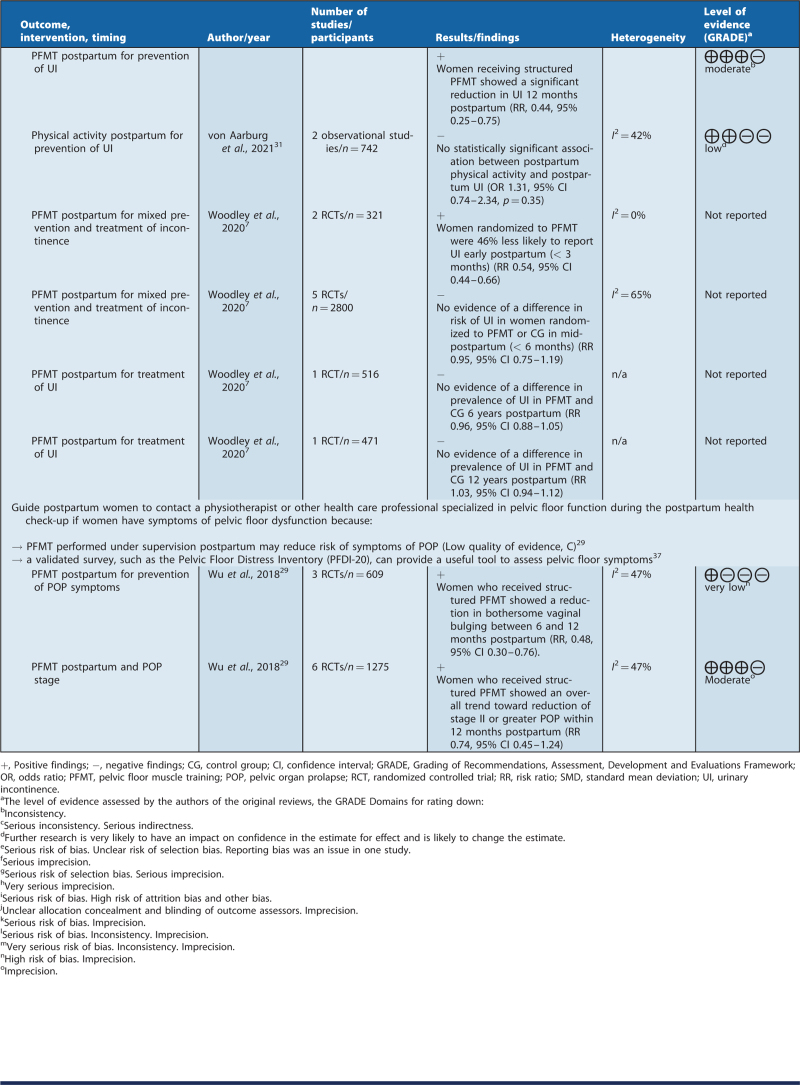
Recommendations for clinical practice and summary of findings

## Discussion

This umbrella review summarizes the results of 9 previous systematic reviews to synthesize knowledge about the effectiveness of exercise interventions on the symptoms of urinary incontinence and POP in pregnant and postpartum women. This review was done as a part of the development of the national clinical practice guideline, “Pregnant and postpartum women in health care: justification and guidance for physical activity and exercise” and the recommendations of the guideline section “Exercise, guided PFMT and function of the pelvic floor” are based on this umbrella review and are presented in this article. Based on our findings, exercise and PFMT can be recommended for pregnant and postpartum women because they prevent and treat urinary incontinence and POP symptoms. The highest level of evidence was found on preventing the symptoms of postpartum urinary incontinence through exercise and PFMT during pregnancy.^7,^^28,^^32–^^34^

Women are encouraged to be physically active and to do aerobic exercise and muscle strengthening activities, including PFMT, to prevent and treat symptoms of the dysfunctions of PFM.^38–^^40^ Interestingly, even though exercise is recommended, the interventions in the included reviews^29,^^30,^^33–^^35^ focused mainly on PFMT. This is surprising, because it is known that doing solely PFMT does not guarantee that women will be able to control the PFM, for example, while exercising or doing daily activities. In future research, it would be important to include PFMT as one of the key elements alongside more versatile exercise. Furthermore, because women are encouraged to do PFMT, it would be important to ensure proper training techniques are provided,^38–^^40^ either through checking with palpation or utilizing biofeedback.^40–^^42^ The NICE guideline^40^ states that supervision of PFMT should involve assessing the woman's ability to perform a pelvic floor contraction and relaxation. Therefore, it is recommended that women who already have symptoms of pelvic floor dysfunction should receive more individualized guidance.^43,^^44^

To identify women who need more individualized guidance, it is recommended to implement a validated Pelvic Floor Distress Inventory (PFDI)-20 form^37^ in maternity care. With the permission of the instrument developers, PFDI-20 was translated into Finnish and is published alongside this guideline. Before this guideline, there was no systematic way to identify those who already have symptoms of pelvic floor dysfunction. As the aim is to identify and treat these women more effectively, it remains important to increase knowledge that symptoms of urinary incontinence and POP are not contra-indicators for exercise and, with the help of individual guidance and knowledge about PFM, exercise is safe and recommended.

### Limitations of the review

The main limitations of this review are the quality of the original studies included in the reviews^23^ and that a meta-analysis could not be conducted because of the heterogeneity of the included reviews. Therefore, grading the level of evidence was also difficult. While grading, the strengths and weaknesses of the evidence were evaluated compared with the GRADE criteria^22^ and the final levels were defined by the clinical guideline working group. In this review, we wanted to include any type of physical activity, including exercise, guidance, physiotherapy, or physiotherapeutic guidance, aiming to address the symptoms of urinary incontinence and POP. As a result, the interventions varied from everyday physical activity, to PFMT, and to different forms of exercise, ranging from strength training to stretching. Due to the variability of the interventions in the included reviews, it was not possible to identify specific recommendations related to the frequency, intensity, or type of exercise. Other limitations are the lack in reporting adherence with the exercise and description of control group. Adherence to exercise was not reported consistently in the original studies even though it is acknowledged that exercise adherence affects treatment outcomes and the effectiveness of the intervention.^45^ In addition, to understand the effectiveness of an intervention, the control group needs to be defined in more detail.

## Conclusion

Based on the findings of this umbrella review, exercise and PFMT can be recommended for pregnant and postpartum women for preventing and treating the common symptoms of urinary incontinence and POP. The highest level of evidence was found within preventing postpartum urinary incontinence symptoms with exercise and PFMT during pregnancy.

### Implications for practice

This review is part of a national clinical practice guideline, which is intended for all social and health care professionals working with pregnant and postpartum women, especially nursing professionals working in maternity care and child health clinics. Evidence transfer and nationwide implementation of the guideline means that the competence of employees must be ensured through education and active dissemination by nurse leaders.^46^ The guideline has already been published on the internet site of the NRF and in a health library that is widely used by health care professionals all over Finland^47^; however, this umbrella review has not been published before.

Nurse leaders in maternity care have a key role in analyzing the barriers and facilitators of guideline implementation in their context. For implementation to be successful, they need to facilitate the change systematically and plan the evaluation process. The next step in evidence transfer and implementation of the guideline is to integrate the recommendations into the Finnish clinical decision support system, EBMEDS.^48^

This guideline should be utilized while resourcing and planning maternity care. In addition to nursing, maternity and child health care clinics require competence in physiotherapy and physical education counseling.

The guideline should be implemented also in social and health care education in the degree programs of nurses, midwives, and physiotherapists. The guideline should be considered while planning continuous education. The guideline can also be used as a reliable source of information for pregnant and postpartum women and their families.^21^

### Implications for research

There is a need for high-quality RCTs where exercise adherence is monitored. Electronic devices could provide a possibility to monitor exercise adherence and intensity as well as supporting adherence by providing feedback. In addition, the term “usual care” should be defined to gain insights into the setting of the research and to better understand effectiveness. The aim of the guideline was to unify the exercise guidance given to pregnant and postpartum women in health care and to reduce unwarranted variations. In the future, it would be important to study the implementation and the use of the guideline in maternity care.

## Acknowledgments

Finnish National Clinical Practice Guideline, “Pregnant and postpartum women in health care: justification and guidance for physical activity,” coordinated by the Nursing Research Foundation, was published in September 2022. This study has been conducted as part of developing the guideline. We are grateful to the guideline working group and the NRF for feedback and guidance.

### Availability of data and materials

The data extraction table and quality assessments are available from the authors upon reasonable request.

### Funding

Translation check of the manuscript was funded by the Nursing Research Foundation.

### Author contributions

All authors have contributed significantly and agree with the content of the manuscript. Author contributions according to the criteria recommended by the ICMJE are: substantial contributions to the conception or design of the work (IR, LH, AA); acquisition (IR, LH), analysis (IR, LH, AA), or interpretation of data (IR, LH, AA); drafting the manuscript (IR, LH) or revising it critically for important intellectual content (LH, HP, AA, AH); final approval of the version to be published (IR, LH, HP, AA, AH); agreement to be accountable for all aspects of the work in ensuring that questions related to the accuracy or integrity of any part of the work are appropriately investigated and resolved (IR, LH, HP, AA, AH).

## Conflict of interest

The authors report no conflicts of interest.

## Supplementary Material
