# Supplementary material for: Effectiveness of exercise interventions on urinary incontinence and pelvic organ prolapse in pregnant and postpartum women: umbrella review and clinical guideline development
Source: JBI Evid Implement. 2023 Oct 18;21(4):394–408. doi: 10.1097/XEB.0000000000000391 (PMC10715701; doi:10.1097/XEB.0000000000000391)
Supplement: Supplementary file 1 [file jbeim-21-394-s001.docx]

**Appendix I: Search terms and strategies**

*Search terms*

| Population | pregnancy, pregnant, nulliparous, prenatal, maternal, antenatal, pregnancy, pregnant, nulliparous, prenatal, maternal, postpartum, postnatal, after delivery, after cesarean section, cesarean section, labor, delivery |
| --- | --- |
| Intervention | physical activity, exercise, movement, motor activity, physiotherapy, rehabilitation, pelvic muscle exercises, yoga, pelvic floor muscle training, guidance, guidelines, health promotion, counselling, exercise, therapy, support, education, patient education, prevention, management |
| Comparison | usual care, no-intervention |
| Outcome | pelvic floor dysfunction, pelvic floor muscle syndrome, overactive pelvic floor, myofascial pelvic pain, levator tension myalgia, hypertonic pelvic floor muscles, pelvic health, pelvic floor disorder, pelvic girdle pain, pelvic girdle function, pelvic girdle dysfunction, cystocele, urethrocele, rectocele, cystourethrocele, enterocele, anterior vaginal wall prolapse, pelvic organ prolapse, posterior vaginal wall prolapse, pelvic fullness, pubocervical vesical fascia weakness, pelvic pressure, vaginal fullness, vaginal pressure, urinary incontinence |

**MEDLINE (PubMed)**

(((((((((("Pregnancy"[Majr]) OR "Pregnant Women"[Majr]))) OR (pregnant[Title] OR pregnancy[Title] OR nulliparous[Title] OR prenatal[Title] OR maternal[Title] OR antenatal[Title]) OR ("Postpartum Period"[Majr]) OR (postpartum[Title] OR postnatal[Title] OR "after delivery"[Title] OR "cesarean section"[Title] OR labour[Title] OR delivery[Title]))))) AND (("Pelvic Floor Disorders"[Majr] OR "Pelvic Girdle Pain"[Majr] OR "Rectocele"[Majr] OR "Pelvic Organ Prolapse"[Majr] OR "Urinary Incontinence"[Majr]) OR (“pelvic floor dysfunction”[Title] OR “pelvic floor muscle dysfunction”[Title] OR "pelvic floor muscle syndrome"[Title] OR “overactive pelvic floor”[Title] OR “myofascial pelvic pain”[Title] OR "levator tension myalgia"[Title] OR “pelvic floor tension myalgia”[Title] OR “hypertonic pelvic floor muscles”[Title] OR “pelvic health”[Title] OR “pelvic floor disorder”[Title] OR “pelvic girdle pain”[Title] OR “symphysis pubis dysfunction”[Title] OR “pelvic girdle function”[Title] OR “pelvic girdle dysfunction”[Title] OR cystocele[Title] OR urethrocele[Title] OR rectocele[Title] OR cystourethrocele[Title] OR enterocele[Title] OR “anterior vaginal wall prolapse”[Title] OR “pelvic organ prolapse”[Title] OR “posterior vaginal wall prolapse”[Title] OR “pelvic fullness”[Title] OR “pubocervical vesical fascia weakness”[Title] OR “pelvic pressure”[Title] OR “vaginal fullness”[Title] OR “vaginal pressure”[Title] OR “urinary incontinence”[Title]))) AND ((((("Exercise"[Majr] OR "Motor Activity"[Majr] OR "Yoga"[Majr])) OR (("physical activity"[Title] OR movement[Title] OR “motor activity”[Title] OR exercise*[Title] OR "physical exercise"[Title] OR “physical training”[Title] OR “exercise training”[Title] OR yoga[Title] OR “pelvic muscle exercise”[Title] OR "pelvic floor muscle training”[Title])) OR "Rehabilitation"[Majr]) OR ((rehabilitation[Title] OR “physical therapy”[Title] OR physiotherapy[Title])))

(((((((((((("Pregnancy"[Majr]) OR "Pregnant Women"[Majr]))) OR (pregnant[Title] OR pregnancy[Title] OR nulliparous[Title] OR prenatal[Title] OR maternal[Title] OR antenatal[Title]) OR ("Postpartum Period"[Majr]) OR (postpartum[Title] OR postnatal[Title] OR "after delivery"[Title] OR "cesarean section"[Title] OR labour[Title] OR delivery[Title]))))) AND (("Pelvic Floor Disorders"[Majr] OR "Pelvic Girdle Pain"[Majr] OR "Rectocele"[Majr] OR "Pelvic Organ Prolapse"[Majr] OR "Urinary Incontinence"[Majr]) OR (“pelvic floor dysfunction”[Title] OR “pelvic floor muscle dysfunction”[Title] OR "pelvic floor muscle syndrome"[Title] OR “overactive pelvic floor”[Title] OR “myofascial pelvic pain”[Title] OR "levator tension myalgia"[Title] OR “pelvic floor tension myalgia”[Title] OR “hypertonic pelvic floor muscles”[Title] OR “pelvic health”[Title] OR “pelvic floor disorder”[Title] OR “pelvic girdle pain”[Title] OR “symphysis pubis dysfunction”[Title] OR “pelvic girdle function”[Title] OR “pelvic girdle dysfunction”[Title] OR cystocele[Title] OR urethrocele[Title] OR rectocele[Title] OR cystourethrocele[Title] OR enterocele[Title] OR “anterior vaginal wall prolapse”[Title] OR “pelvic organ prolapse”[Title] OR “posterior vaginal wall prolapse”[Title] OR “pelvic fullness”[Title] OR “pubocervical vesical fascia weakness”[Title] OR “pelvic pressure”[Title] OR “vaginal fullness”[Title] OR “vaginal pressure”[Title] OR “urinary incontinence”[Title]))) AND ((((("Exercise"[Majr] OR "Motor Activity"[Majr] OR "Yoga"[Majr])) OR (("physical activity"[Title] OR movement[Title] OR “motor activity”[Title] OR exercise*[Title] OR "physical exercise"[Title] OR “physical training”[Title] OR “exercise training”[Title] OR yoga[Title] OR “pelvic muscle exercise”[Title] OR "pelvic floor muscle training”[Title])) OR "Rehabilitation"[Majr]) OR ((rehabilitation[Title] OR “physical therapy”[Title] OR physiotherapy[Title]))))) AND ((("Guidelines as Topic"[Majr] OR "Health Promotion"[Majr] OR "Counseling"[Majr] OR "Exercise Therapy"[Majr] OR "Patient Education as Topic"[Majr])) OR (guidelines[Title] OR guidance[Title] OR “health promotion”[Title] OR counsel*[Title] OR support*[Title] OR “exercise therapy”[Title] OR education[Title] OR “patient education”[Title] OR prevention[Title] OR management[Title]))

**CINAHL (EBSCOhost)**

TI (pregnant OR pregnancy OR nulliparous OR prenatal OR maternal OR antenatal OR postpartum OR postnatal OR "after delivery" OR "cesarean section" OR labour OR delivery) AND TI (“pelvic floor dysfunction” OR “pelvic floor muscle dysfunction” OR "pelvic floor muscle syndrome" OR “overactive pelvic floor” OR “myofascial pelvic pain” OR "levator tension myalgia" OR “pelvic floor tension myalgia” OR “hypertonic pelvic floor muscles” OR “pelvic health” OR “pelvic floor disorder*” OR “pelvic girdle pain” OR “symphysis pubis dysfunction” OR “pelvic girdle function” OR “pelvic girdle dysfunction” OR cystocele OR urethrocele OR rectocele OR cystourethrocele OR enterocele OR “anterior vaginal wall prolapse” OR “pelvic organ prolapse” OR “posterior vaginal wall prolapse” OR “pelvic fullness” OR “pubocervical vesical fascia weakness” OR “pelvic pressure” OR “vaginal fullness” OR “vaginal pressure” OR “urinary incontinence”) AND TI ("physical activity" OR movement OR “motor activity” OR exercise* OR "physical exercise" OR “physical training” OR “exercise training” OR yoga OR “pelvic muscle exercise” OR "pelvic floor muscle training” OR rehabilitation OR “physical therapy” OR physiotherapy)

TI (pregnant OR pregnancy OR nulliparous OR prenatal OR maternal OR antenatal OR postpartum OR postnatal OR "after delivery" OR "cesarean section" OR labour OR delivery) AND TI (“pelvic floor dysfunction” OR “pelvic floor muscle dysfunction” OR "pelvic floor muscle syndrome" OR “overactive pelvic floor” OR “myofascial pelvic pain” OR "levator tension myalgia" OR “pelvic floor tension myalgia” OR “hypertonic pelvic floor muscles” OR “pelvic health” OR “pelvic floor disorder*” OR “pelvic girdle pain” OR “symphysis pubis dysfunction” OR “pelvic girdle function” OR “pelvic girdle dysfunction” OR cystocele OR urethrocele OR rectocele OR cystourethrocele OR enterocele OR “anterior vaginal wall prolapse” OR “pelvic organ prolapse” OR “posterior vaginal wall prolapse” OR “pelvic fullness” OR “pubocervical vesical fascia weakness” OR “pelvic pressure” OR “vaginal fullness” OR “vaginal pressure” OR “urinary incontinence”) AND TI ("physical activity" OR movement OR “motor activity” OR exercise* OR "physical exercise" OR “physical training” OR “exercise training” OR yoga OR “pelvic muscle exercise” OR "pelvic floor muscle training” OR rehabilitation OR “physical therapy” OR physiotherapy) AND TI (guideline* OR guidance OR “health promotion” OR counsel* OR support* OR “exercise therapy” OR education OR “patient education” OR prevention OR management)

**PsycINFO (EBSCOhost)**

TI (pregnant OR pregnancy OR nulliparous OR prenatal OR maternal OR antenatal OR postpartum OR postnatal OR "after delivery" OR "cesarean section" OR labour OR delivery) AND TI (“pelvic floor dysfunction” OR “pelvic floor muscle dysfunction” OR "pelvic floor muscle syndrome" OR “overactive pelvic floor” OR “myofascial pelvic pain” OR "levator tension myalgia" OR “pelvic floor tension myalgia” OR “hypertonic pelvic floor muscles” OR “pelvic health” OR “pelvic floor disorder*” OR “pelvic girdle pain” OR “symphysis pubis dysfunction” OR “pelvic girdle function” OR “pelvic girdle dysfunction” OR cystocele OR urethrocele OR rectocele OR cystourethrocele OR enterocele OR “anterior vaginal wall prolapse” OR “pelvic organ prolapse” OR “posterior vaginal wall prolapse” OR “pelvic fullness” OR “pubocervical vesical fascia weakness” OR “pelvic pressure” OR “vaginal fullness” OR “vaginal pressure” OR “urinary incontinence”) AND TI ("physical activity" OR movement OR “motor activity” OR exercise* OR "physical exercise" OR “physical training” OR “exercise training” OR yoga OR “pelvic muscle exercise” OR "pelvic floor muscle training” OR rehabilitation OR “physical therapy” OR physiotherapy)

TI (pregnant OR pregnancy OR nulliparous OR prenatal OR maternal OR antenatal OR postpartum OR postnatal OR "after delivery" OR "cesarean section" OR labour OR delivery) AND TI (“pelvic floor dysfunction” OR “pelvic floor muscle dysfunction” OR "pelvic floor muscle syndrome" OR “overactive pelvic floor” OR “myofascial pelvic pain” OR "levator tension myalgia" OR “pelvic floor tension myalgia” OR “hypertonic pelvic floor muscles” OR “pelvic health” OR “pelvic floor disorder*” OR “pelvic girdle pain” OR “symphysis pubis dysfunction” OR “pelvic girdle function” OR “pelvic girdle dysfunction” OR cystocele OR urethrocele OR rectocele OR cystourethrocele OR enterocele OR “anterior vaginal wall prolapse” OR “pelvic organ prolapse” OR “posterior vaginal wall prolapse” OR “pelvic fullness” OR “pubocervical vesical fascia weakness” OR “pelvic pressure” OR “vaginal fullness” OR “vaginal pressure” OR “urinary incontinence”) AND TI ("physical activity" OR movement OR “motor activity” OR exercise* OR "physical exercise" OR “physical training” OR “exercise training” OR yoga OR “pelvic muscle exercise” OR "pelvic floor muscle training” OR rehabilitation OR “physical therapy” OR physiotherapy) AND TI (guideline* OR guidance OR “health promotion” OR counsel* OR support* OR “exercise therapy” OR education OR “patient education” OR prevention OR management)

**Web of Science**

postnatal OR "after delivery" OR "cesarean section" OR labour OR delivery) AND TI=(“pelvic floor dysfunction” OR “pelvic floor muscle dysfunction” OR "pelvic floor muscle syndrome" OR “overactive pelvic floor” OR “myofascial pelvic pain” OR "levator tension myalgia" OR “pelvic floor tension myalgia” OR “hypertonic pelvic floor muscles” OR “pelvic health” OR “pelvic floor disorder*” OR “pelvic girdle pain” OR “symphysis pubis dysfunction” OR “pelvic girdle function” OR “pelvic girdle dysfunction” OR cystocele OR urethrocele OR rectocele OR cystourethrocele OR enterocele OR “anterior vaginal wall prolapse” OR “pelvic organ prolapse” OR “posterior vaginal wall prolapse” OR “pelvic fullness” OR “pubocervical vesical fascia weakness” OR “pelvic pressure” OR “vaginal fullness” OR “vaginal pressure” OR “urinary incontinence”) AND TI=("physical activity" OR movement OR “motor activity” OR exercise* OR "physical exercise" OR “physical training” OR “exercise training” OR yoga OR “pelvic muscle exercise” OR "pelvic floor muscle training” OR rehabilitation OR “physical therapy” OR physiotherapy)

TI=(pregnant OR pregnancy OR nulliparous OR prenatal OR maternal OR antenatal OR postpartum OR postnatal OR "after delivery" OR "cesarean section" OR labour OR delivery) AND TI=(“pelvic floor dysfunction” OR “pelvic floor muscle dysfunction” OR "pelvic floor muscle syndrome" OR “overactive pelvic floor” OR “myofascial pelvic pain” OR "levator tension myalgia" OR “pelvic floor tension myalgia” OR “hypertonic pelvic floor muscles” OR “pelvic health” OR “pelvic floor disorder*” OR “pelvic girdle pain” OR “symphysis pubis dysfunction” OR “pelvic girdle function” OR “pelvic girdle dysfunction” OR cystocele OR urethrocele OR rectocele OR cystourethrocele OR enterocele OR “anterior vaginal wall prolapse” OR “pelvic organ prolapse” OR “posterior vaginal wall prolapse” OR “pelvic fullness” OR “pubocervical vesical fascia weakness” OR “pelvic pressure” OR “vaginal fullness” OR “vaginal pressure” OR “urinary incontinence”) AND TI=("physical activity" OR movement OR “motor activity” OR exercise* OR "physical exercise" OR “physical training” OR “exercise training” OR yoga OR “pelvic muscle exercise” OR "pelvic floor muscle training” OR rehabilitation OR “physical therapy” OR physiotherapy) AND TI=(guideline* OR guidance OR “health promotion” OR counsel* OR support* OR “exercise therapy” OR education OR “patient education” OR prevention OR management)

**Medic**

***Te/Ot/As/Ti*** (pregnancy pregnant postpartum raskau* raskaa* synnyttän*) AND (“pelvic floor” “pelvic girdle” “vaginal wall prolapse” lantionpohja* lantiorenka* laskeum*) AND (exercise* ”physical activity” ”physical training” yoga* liikunta* liikunn* liikuntaharjoittelu* liikkumi* ”fyysinen aktiivisuus” jooga* physiotherap* “physical therapy” counsel* support* fysioterap* ohjau* neuvon* tukem*)

**Cochrane Library**

(Pregnanc* OR Pregnant NEXT Women OR Postpartum NEXT Period OR nulliparous OR prenatal OR maternal OR antenatal OR postpartum OR postnatal OR after NEXT delivery OR cesarean NEXT section OR labour OR delivery) AND (Pelvic NEXT Floor NEXT Disorders OR Pelvic NEXT Girdle NEXT Pain OR Rectocele OR Pelvic NEXT Organ NEXT Prolapse OR Urinary NEXT Incontinence OR pelvic NEXT floor NEXT muscle NEXT dysfunction OR pelvic NEXT floor NEXT muscle NEXT syndrome OR overactive NEXT pelvic NEXT floor OR myofascial NEXT pelvic NEXT pain OR levator NEXT tension NEXT myalgia OR pelvic NEXT floor NEXT tension NEXT myalgia OR hypertonic NEXT pelvic NEXT floor NEXT muscles OR pelvic NEXT health OR pelvic NEXT floor NEXT disorder OR pelvic NEXT girdle NEXT pain OR symphysis NEXT pubis NEXT dysfunction OR pelvic NEXT girdle NEXT function OR pelvic NEXT girdle NEXT dysfunction OR cystocele OR urethrocele OR rectocele OR cystourethrocele OR enterocele OR anterior NEXT vaginal NEXT wall NEXT prolapse OR pelvic NEXT organ NEXT prolapse OR posterior NEXT vaginal NEXT wall NEXT prolapse OR pelvic NEXT fullness OR pubocervical NEXT vesical NEXT fascia NEXT weaknes* OR pelvic NEXT pressure OR vaginal NEXT fullness OR vaginal NEXT pressure OR urinary NEXT incontinence) AND (Exercise OR Motor NEXT Activity OR Yoga OR Rehabilitation OR physical NEXT activity OR movement OR physical NEXT exercise OR physical NEXT training OR exercise NEXT training OR pelvic NEXT muscle NEXT exercise OR pelvic NEXT floor NEXT muscle NEXT training OR physical NEXT therapy OR physiotherapy)

**ERIC (EBSCOhost)**

TI (pregnant OR pregnancy OR nulliparous OR prenatal OR maternal OR antenatal OR postpartum OR postnatal OR "after delivery" OR "cesarean section" OR labour OR delivery) AND TI (“pelvic floor dysfunction” OR “pelvic floor muscle dysfunction” OR "pelvic floor muscle syndrome" OR “overactive pelvic floor” OR “myofascial pelvic pain” OR "levator tension myalgia" OR “pelvic floor tension myalgia” OR “hypertonic pelvic floor muscles” OR “pelvic health” OR “pelvic floor disorder*” OR “pelvic girdle pain” OR “symphysis pubis dysfunction” OR “pelvic girdle function” OR “pelvic girdle dysfunction” OR cystocele OR urethrocele OR rectocele OR cystourethrocele OR enterocele OR “anterior vaginal wall prolapse” OR “pelvic organ prolapse” OR “posterior vaginal wall prolapse” OR “pelvic fullness” OR “pubocervical vesical fascia weakness” OR “pelvic pressure” OR “vaginal fullness” OR “vaginal pressure” OR “urinary incontinence”) AND TI ("physical activity" OR movement OR “motor activity” OR exercise* OR "physical exercise" OR “physical training” OR “exercise training” OR yoga OR “pelvic muscle exercise” OR "pelvic floor muscle training” OR rehabilitation OR “physical therapy” OR physiotherapy)

TI (pregnant OR pregnancy OR nulliparous OR prenatal OR maternal OR antenatal OR postpartum OR postnatal OR "after delivery" OR "cesarean section" OR labour OR delivery) AND TI (“pelvic floor dysfunction” OR “pelvic floor muscle dysfunction” OR "pelvic floor muscle syndrome" OR “overactive pelvic floor” OR “myofascial pelvic pain” OR "levator tension myalgia" OR “pelvic floor tension myalgia” OR “hypertonic pelvic floor muscles” OR “pelvic health” OR “pelvic floor disorder*” OR “pelvic girdle pain” OR “symphysis pubis dysfunction” OR “pelvic girdle function” OR “pelvic girdle dysfunction” OR cystocele OR urethrocele OR rectocele OR cystourethrocele OR enterocele OR “anterior vaginal wall prolapse” OR “pelvic organ prolapse” OR “posterior vaginal wall prolapse” OR “pelvic fullness” OR “pubocervical vesical fascia weakness” OR “pelvic pressure” OR “vaginal fullness” OR “vaginal pressure” OR “urinary incontinence”) AND TI ("physical activity" OR movement OR “motor activity” OR exercise* OR "physical exercise" OR “physical training” OR “exercise training” OR yoga OR “pelvic muscle exercise” OR "pelvic floor muscle training” OR rehabilitation OR “physical therapy” OR physiotherapy) AND TI (guideline* OR guidance OR “health promotion” OR counsel* OR support* OR “exercise therapy” OR education OR “patient education” OR prevention OR management)

**Embase**

(pregnant OR pregnancy OR nulliparous OR prenatal OR maternal OR antenatal OR postpartum OR postnatal OR "after delivery" OR "cesarean section" OR labour OR delivery):TI AND (“pelvic floor dysfunction” OR “pelvic floor muscle dysfunction” OR "pelvic floor muscle syndrome" OR “overactive pelvic floor” OR “myofascial pelvic pain” OR "levator tension myalgia" OR “pelvic floor tension myalgia” OR “hypertonic pelvic floor muscles” OR “pelvic health” OR “pelvic floor disorder*” OR “pelvic girdle pain” OR “symphysis pubis dysfunction” OR “pelvic girdle function” OR “pelvic girdle dysfunction” OR cystocele OR urethrocele OR rectocele OR cystourethrocele OR enterocele OR “anterior vaginal wall prolapse” OR “pelvic organ prolapse” OR “posterior vaginal wall prolapse” OR “pelvic fullness” OR “pubocervical vesical fascia weakness” OR “pelvic pressure” OR “vaginal fullness” OR “vaginal pressure” OR “urinary incontinence”):TI AND ("physical activity" OR movement OR “motor activity” OR exercise* OR "physical exercise" OR “physical training” OR “exercise training” OR yoga OR “pelvic muscle exercise” OR "pelvic floor muscle training” OR rehabilitation OR “physical therapy” OR physiotherapy):TI

**Academic Search Premier (EBSCOhost)**

TI (pregnant OR pregnancy OR nulliparous OR prenatal OR maternal OR antenatal OR postpartum OR postnatal OR "after delivery" OR "cesarean section" OR labour OR delivery) AND TI (“pelvic floor dysfunction” OR “pelvic floor muscle dysfunction” OR "pelvic floor muscle syndrome" OR “overactive pelvic floor” OR “myofascial pelvic pain” OR "levator tension myalgia" OR “pelvic floor tension myalgia” OR “hypertonic pelvic floor muscles” OR “pelvic health” OR “pelvic floor disorder*” OR “pelvic girdle pain” OR “symphysis pubis dysfunction” OR “pelvic girdle function” OR “pelvic girdle dysfunction” OR cystocele OR urethrocele OR rectocele OR cystourethrocele OR enterocele OR “anterior vaginal wall prolapse” OR “pelvic organ prolapse” OR “posterior vaginal wall prolapse” OR “pelvic fullness” OR “pubocervical vesical fascia weakness” OR “pelvic pressure” OR “vaginal fullness” OR “vaginal pressure” OR “urinary incontinence”) AND TI ("physical activity" OR movement OR “motor activity” OR exercise* OR "physical exercise" OR “physical training” OR “exercise training” OR yoga OR “pelvic muscle exercise” OR "pelvic floor muscle training” OR rehabilitation OR “physical therapy” OR physiotherapy)

TI (pregnant OR pregnancy OR nulliparous OR prenatal OR maternal OR antenatal OR postpartum OR postnatal OR "after delivery" OR "cesarean section" OR labour OR delivery) AND TI (“pelvic floor dysfunction” OR “pelvic floor muscle dysfunction” OR "pelvic floor muscle syndrome" OR “overactive pelvic floor” OR “myofascial pelvic pain” OR "levator tension myalgia" OR “pelvic floor tension myalgia” OR “hypertonic pelvic floor muscles” OR “pelvic health” OR “pelvic floor disorder*” OR “pelvic girdle pain” OR “symphysis pubis dysfunction” OR “pelvic girdle function” OR “pelvic girdle dysfunction” OR cystocele OR urethrocele OR rectocele OR cystourethrocele OR enterocele OR “anterior vaginal wall prolapse” OR “pelvic organ prolapse” OR “posterior vaginal wall prolapse” OR “pelvic fullness” OR “pubocervical vesical fascia weakness” OR “pelvic pressure” OR “vaginal fullness” OR “vaginal pressure” OR “urinary incontinence”) AND TI ("physical activity" OR movement OR “motor activity” OR exercise* OR "physical exercise" OR “physical training” OR “exercise training” OR yoga OR “pelvic muscle exercise” OR "pelvic floor muscle training” OR rehabilitation OR “physical therapy” OR physiotherapy) AND TI (guideline* OR guidance OR “health promotion” OR counsel* OR support* OR “exercise therapy” OR education OR “patient education” OR prevention OR management)
